# Health economic analysis of human papillomavirus vaccines in women of Chile: perspective of the health care payer using a Markov model

**DOI:** 10.1186/1471-2458-14-1222

**Published:** 2014-11-26

**Authors:** Jorge Alberto Gomez, Alejandro Lepetic, Nadia Demarteau

**Affiliations:** GSK Vaccines Latin America, Carlos Casares, 3690, B1644CD Victoria, Buenos Aires, Argentina; GSK Vaccines, Wavre, Belgium

**Keywords:** HPV vaccines, Economic evaluation, Chile, Cervical cancer, Genital warts, Low-risk, Screening

## Abstract

**Background:**

In Chile, significant reductions in cervical cancer incidence and mortality have been observed due to implementation of a well-organized screening program. However, it has been suggested that the inclusion of human papillomavirus (HPV) vaccination for young adolescent women may be the best prospect to further reduce the burden of cervical cancer. This cost-effectiveness study comparing two available HPV vaccines in Chile was performed to support decision making on the implementation of universal HPV vaccination.

**Methods:**

The present analysis used an existing static Markov model to assess the effect of screening and vaccination. This analysis includes the epidemiology of low-risk HPV types allowing for the comparison between the two vaccines (HPV-16/18 AS04-adjuvanted vaccine and the HPV-6/11/16/18 vaccine), latest cross-protection data on HPV vaccines, treatment costs for cervical cancer, vaccine costs and 6% discounting per the health economic guideline for Chile.

**Results:**

Projected incremental cost-utility ratio (ICUR) and incremental cost-effectiveness ratio (ICERs) for the HPV-16/18 AS04-adjuvanted vaccine was 116 United States (US) dollars per quality-adjusted life years (QALY) gained or 147 US dollars per life-years (LY) saved, while the projected ICUR/ICER for the HPV-6/11/16/18 vaccine was 541 US dollars per QALY gained or 726 US dollars per LY saved. Introduction of any HPV vaccine to the present cervical cancer prevention program of Chile is estimated to be highly cost-effective (below 1X gross domestic product [GDP] per capita, 14278 US dollars). In Chile, the addition of HPV-16/18 AS04-adjuvanted vaccine to the existing screening program dominated the addition of HPV-6/11/16/18 vaccine. In the probabilistic sensitivity analysis results show that the HPV-16/18 AS04-adjuvanted vaccine is expected to be dominant and cost-saving in 69.3% and 77.6% of the replicates respectively.

**Conclusions:**

The findings indicate that the addition of any HPV vaccine to the current cervical screening program of Chile will be advantageous. However, this cost-effectiveness model shows that the HPV-16/18 AS04-adjuvanted vaccine dominated the HPV-6/11/16/18 vaccine. Beyond the context of Chile, the data from this modelling exercise may support healthcare policy and decision-making pertaining to introduction of HPV vaccination in similar resource settings in the region.

**Electronic supplementary material:**

The online version of this article (doi:10.1186/1471-2458-14-1222) contains supplementary material, which is available to authorized users.

## Background

Cervical cancer is the second most common cancer in women of the Latin America and Caribbean (LAC) region with estimated age-standardized incidence and mortality rates of 23.5 and 10.8 per 100,000 women, respectively [1]. Organized screening programs based on cytology have reduced the cervical cancer burden through early detection and treatment of pre-cancerous lesions [2, 3]. Very few countries from the LAC region have shown a slight decrease in cervical cancer disease burden following implementation of screening programmes [4, 5]. This may be due in part to difficulties in the implementation of organized screening programs in these low-resource settings. Moreover, ensuring utilization of services provided through these screening programs poses an additional challenge in this region. There is great disparity in the approach between countries to reduce the burden of cervical cancer in the LAC region. Countries like Chile have a well-organized screening program and therefore low cervical cancer morbidity and mortality compared to other countries in the LAC region. Chile is one of the few countries in this region with a functional organized screening program [6]. A 162% increase in Papanicolaou’s (Pap) smear testing coverage (1990–2004), and a 48.0% decrease in cervical cancer mortality (1987–2003) were observed in Chile after the implementation of the screening programme [7, 8]. In most of the other countries in the LAC region where no screening programs exist, the incidence and mortality associated with cervical cancer remain high [1].

New opportunities to tackle this high burden of cervical cancer disease are now available. Two different vaccines against human papillomavirus (HPV) have been widely used, both internationally and in the LAC region [3, 9]. It is expected that these vaccines if combined with existing screening programs could become a critical component of cervical cancer prevention programs [9].

Cost-effectiveness analysis of new interventions, like the introduction of HPV vaccines is a useful tool to assist decision makers in allocating resources. Results reported from these analyses vary widely from one country to another due to epidemiological variation, treatment-related costs and effectiveness of existing secondary prevention programs. The potential impact and cost-effectiveness of introducing the bivalent HPV-16/18 AS04-adjuvanted vaccine to the cervical cancer prevention programs of five Latin American countries was previously evaluated [10]. That analysis has shown that the incremental cost-effectiveness ratio (ICERs) was higher for Chile where a successful screening program existed [10]. For countries like Chile it is therefore more difficult to achieve a good and robust cost-effectiveness profile.

HPV vaccines have now been offered to Latin American countries through the Pan American Health Organization (PAHO) Revolving Fund at low prices per vaccine dose following which some countries in the LAC region have decided its inclusion in their national vaccination programs (Panama, Mexico, Peru and Argentina) [11]. The present cost-effectiveness and cost-utility analysis was performed to assess the feasibility of including a HPV vaccination program to the current screening program in Chile while updating the original model and input parameters based on availability of new data [10]. This analysis includes the latest cross-protection data for both HPV vaccines, up to-date treatment costs for cervical cancer generated by the Ministry of Health of Chile, including specific health economic recommendations for Chile on discounting, vaccine prices per dose for the public health sector and the epidemiology of low-risk HPV types (HPV types that rarely cause cancer and include HPV types such as 6 and 11) allowing for the comparison between the two HPV vaccines as well as a potential two-dose HPV vaccination schedule in an additional scenario analysis.

## Methods

### Model structure

This analysis used a previously published lifetime Markov cohort model with a one year cycle reproducing the natural history of oncogenic HPV in cervical cancer, the effect of screening and of vaccination [10, 12–15] during the lifetime of the cohort. The model was previously evaluated in terms of its capacity, requirements, limitations and comparability in a study conducted by the World Health Organization [16] and has been extended by including infection with low-risk HPV types that might lead to the development of a cervical intraepithelial neoplasia (CIN) grade 1 (CIN1) lesion and genital warts (GW) [17]. The model structure used to analyze the cost-effectiveness of the HPV-16/18 AS04-adjuvanted vaccine and HPV-6/11/16/18 vaccine for Chile has been described previously [17]. The present model considers the perspective of the healthcare payer thereby allowing for the comparison of three different intervention strategies – current screening program (reference scenario), current screening program and vaccination with the HPV-16/18 AS04-adjuvanted vaccine; and current screening program and vaccination with the HPV-6/11/16/18 vaccine. The model was validated against reported cervical cancer incidence, cervical cancer death and GW incidence.

### Input parameters

Most of the input parameters used to calibrate the model for Chile were previously described [10] and are summarized in Table 1.

**Table 1:** **Input data, base case**

Population data	
*11-years old women cohort (2012)*	123,581 [18]
**Screening characteristics** [19]	
*Regular screening coverage*	57.0% [20]
*Interval between regular screening*	3 years [10]
*Irregular screening coverage*	0.0%
*Population without screening*	43.0% [20]
*Age of initiation of screening*	25 years [10]
*Sensitivity to detect CIN1*	58.0% [10]
*Sensitivity to detect CIN2 and CIN3*	61.0% [10]
*Estimated positive Pap smear*	1.7% [10]
**Treatment performance** [21]	
*CIN1 detected by the screening and undergoing treatment* ^*a*^	40.0% [22]
*Efficacy of CIN1 treatment* ^*a*^	100.0% [10]
*CIN1 treatment effectiveness* ^*b*^	40.0%
*CIN2 and CIN3 detected by the screening and undergoing treatment* ^*a*^	100.0% [10]
*Efficacy of CIN 2 and 3 treatment* ^*a*^	90.0% [10]
*CIN2 and CIN3 treatment effectiveness* ^*b*^	90.0%
*Five-year cancer cure rate*	64.0% [1]
**Parameters to estimate vaccine effectiveness**	
*Prevalence of HPV types 16 and 18 in cervical cancer*	80.0% [23]
*Prevalence of other oncogenic HPV in cervical cancer*	20.0% [23]
*Prevalence of HPV types 16 and 18 in CIN23*	46.5% [19]
*Prevalence of other oncogenic HPV in CIN23*	29.8% [19]
*Prevalence of HPV types 16 and 18 in CIN1*	36.5% [19]
*Prevalence of other oncogenic HPV in CIN1*	42.9% [19]
*Prevalence of HPV types 6 and 11 in CIN1*	15.8% [19]
*Prevalence of HPV types 6 and 11 in GW*	76.2% [24]
*Vaccine efficacy to HPV types16 and 18 CC (HPV-16/18 & HPV-6/11/16/18 vaccines)*	98.0%/98.0% [25–27]
*Vaccine efficacy to HPV types16 and 18 CIN23 (HPV-16/18 & HPV-6/11/16/18 vaccines)*	98.0%/98.0% [25–27]
*Vaccine efficacy to HPV types16 and 18 CIN1 (HPV-16/18 & HPV-6/11/16/18 vaccines)*	98.0%/98.0% [25–27]
*Vaccine efficacy to other oncogenic HPV CC (HPV-16/18 & HPV-6/11/16/18 vaccines)*	68.4%/32.5% [28–31]
*Vaccine efficacy to other oncogenic HPV CIN23 (HPV-16/18 & HPV-6/11/16/18 vaccines)*	68.4%/32.5% [28–31]
*Vaccine efficacy to other oncogenic HPV CIN1 (HPV-16/18 & HPV-6/11/16/18 vaccines)*	47.7%/23.4% [28, 29, 32]
*Vaccine efficacy to HPV types 6 and 11 for HPV-6/11/16/18 vaccine*	98.0% [33, 34]
**Costs** [35]	**US dollars**
*Pap smear cost (including false positive tests)*	26.19
*GW treatment*	62.00
*CIN1 treatment & follow-up cost*	1,636.00
*CIN2 and CIN3 treatment & follow-up cost*	1,636.00
*Cervical cancer treatment*	13,218.00
*Cost per vaccinated woman (price parity between vaccines)* ^*c*^	60.00

^a^Expert opinion; ES; ^b^Calculated; ^c^Price parity at 20 US dollars per dose were used.

Note: CIN: cervical intraepithelial neoplasia; HPV: human papillomavirus; US: United States.

#### Vaccinated population and coverage

The vaccinated cohort assumed in the model included women aged 11 years (n = 123581; population statistics, 2009) [18]. Vaccination coverage was estimated to be 95.0% (complete schedule at 12 years of age); this was based on similar assumptions made by health authorities in Chile [35].

#### Epidemiological data

In the present analysis new or modified epidemiological parameters compared with the previous Chilean adaptation of the model are described. The proportion of CIN1 cases under treatment were reduced to 40.0% following the new clinical guidelines developed by the Ministry of Health, Chile [22]. Five-year cervical cancer cure rate was updated to 64.0%.The coverage of regular screening during 2009 and 2010 was reported to be 56.7% [20]. The age-specific incidence of low-risk HPV was calculated from prevalence data and progression and regression rate; the method used is detailed in Additional file 1[36]. The incidence of GW was calculated based on the number of reported cases in women by the Ministry of Health, Chile (n = 4685; 2008) [35]. Age distribution of GW was matched to data reported in a French study [37] since this was not available for Chile. The transition from low risk HPV infection and GW incidence was estimated from the ratio between these two parameters by age.

#### Medical costs

The costs of treatment of precancerous lesions and cervical cancer were obtained from data published by the Ministry of Health, Chile (Table 1) [35]. Weighted average between reported public and private costs was used and the original costs in Chilean Pesos (2010) were translated to US dollars (2010) (1 US dollar = 469.7 pesos) [38]. Country-specific costs associated with HPV infection and screening were limited to direct medical costs as reported by the Ministry of Health, Chile [35].

#### Vaccine costs

The cost of vaccination was assumed to be 20 US dollars per dose for both vaccines. Although the cost of vaccines is slightly lower through the PAHO Revolving Fund, this value of 20 US dollars per dose was assumed in the analysis since an accurate estimation of the cost of vaccine administration was unavailable. The cost of administration was assumed to be the same for the two vaccines.

#### Vaccine efficacy and cross-protection

In the estimation of vaccine effectiveness, HPV type-specific vaccine efficacies and HPV type prevalence in cervical cancer and precancerous lesions for Chile were considered. Vaccine efficacy of 98.0% against cervical cancer, CIN1, CIN grade 2 (CIN2) and CIN grade 3 (CIN3) associated with HPV types 16 and 18 were assumed to be identical for the HPV-16/18 AS04-adjuvanted vaccine and HPV-6/11/16/18 vaccine [25–27]. Additionally, cross-protection data was used for both vaccines (see Additional file 1). Although there is no study directly comparing the cross-protection benefit associated with the HPV-16/18 AS04-adjuvanted and HPV-6/11/16/18 vaccines, analysis of the most comparable populations from clinical trials of both the vaccines [28–32] suggest that cross-protective vaccine efficacy estimates against infections and lesions associated with non-vaccine HPV types were higher for the HPV-16/18 AS04-adjuvanted vaccines than the HPV-6/11/16/18 vaccine. Cross-protection levels for the HPV-16/18 AS04-adjuvanted vaccine (PATRICIA study) and HPV-6/11/16/18 vaccine (FUTURE I/II studies) against the ten most frequent oncogenic HPV types after HPV types 16 and 18 (including HPV types 31, 33, 35, 39, 45, 51, 52, 56, 58 and 59) were selected in order to use robust estimates [28–32]. These specific cross protection parameters against the same ten non-vaccine and oncogenic HPV types for both vaccines were reported from their respective clinical trials. Cross protection considered against cervical cancer, CIN2 and CIN3 associated with these ten oncogenic HPV types was 68.4% (HPV-16/18 AS04-adjuvanted vaccine) [29–31] and 33.0% (HPV-6/11/16/18 vaccine) [28]. Similarly, cross protection levels against CIN1 associated with these ten oncogenic HPV types were 47.7% (HPV-16/18 AS04-adjuvanted vaccine) [29, 32] and 23.0% (HPV-6/11/16/18 vaccine) [29]. The efficacy of HPV-6/11/16/18 vaccine against low-risk HPV types 6 and 11 was assumed to be 98.0% [33, 34]. The prevalence of HPV types in cervical cancer, CIN1, CIN2 and CIN3 were obtained from different studies conducted in Chile (Table 1) [19, 23].

Lifetime sustained protection against HPV types 16 and 18 was considered in the base case analysis. Based on clinical trial data, efficacy against the HPV-16/18 AS04-adjuvanted vaccine after 6.4 years and 9.4 years of follow-up was observed [21, 26]. Furthermore, modelling studies (based on 6.4 years data) have predicted long-term (≥20 years) persistence of HPV types 16 and 18 antibodies for the HPV-16/18 AS04-adjuvanted vaccine [39]. Therefore, sustained protection was assumed to remain true over a lifetime in the base case analysis and the impact of waning effects on cost-effectiveness results was assessed in additional scenarios (see Additional file 1). As compliance to the recommended vaccine schedule is often not optimal in adolescents, studies conducted in adolescent girls have shown that a two dose schedule of HPV-16/18 AS04-adjuvanted vaccine administered in adolescents aged 9–14 years was non-inferior to a three dose schedule administered to subjects aged 15–25 years up to 24 months post-vaccination [40]. Efficacy estimates of the HPV-16/18 AS04-adjuvanted vaccine by dose in a non-randomized analysis suggested that a two dose schedule of the vaccine may be as protective as a three dose regimen against persistent infection with HPV types 16 and 18 in women [41]. Therefore, a potential two dose schedule for the HPV-16/18 AS04-adjuvanted vaccine was considered as an additional scenario of interest, based on the latest publications of Kreimer et al. [41] and Romanovski et al. [40]. For the two dose HPV vaccination schedule, vaccine efficacies against the different HPV types were assumed to be similar to the three dose schedule.

### Base case analyses

The present study assessed the incremental difference in health outcomes, total costs and ICER (cost per quality-adjusted life years [QALY] gained) between scenarios. Future costs and health outcomes were discounted at an annual rate of 6.0% based on the recommendations of the Planning Ministry of Chile [42] and used by the Ministry of Health, Chile [22]. The effects of discount rate on the outcomes in the different scenarios were assessed. The discounted ICER (discounted cost per QALY gained) estimated for each vaccination scenario was compared to the cost-effectiveness threshold defined by the World Health Organization [43]. Data on gross domestic product (GDP) per capita for Chile (14278 US dollars per capita for 2011) was obtained from the International Monetary Fund [44].

### Deterministic scenario analyses

In addition to the base case scenarios for both vaccines, deterministic scenario analyses were conducted by varying key input parameters of interest:

- Prevalence of HPV types in cervical cancer cases used for base case analysis was obtained from a recent study [23] which reported high prevalence of HPV types 16 and 18 (80.0% assumed for base case scenario). As this value is slightly higher than usually reported prevalence of 70.0% for HPV types 16 and 18 [45], additional scenarios for both the vaccines assuming prevalence of 70.0% for HPV types 16 and 18 and for the other oncogenic HPV types as 30.0% were tested.
- Discount rate of 6.0% was used in base case; however, a discount rate of 3.0% was also tested in-line with international guidelines.
- Introduction of the HPV-16/18 AS04-adjuvanted vaccine to the screening program of Chile at an increased screening interval as a control was considered of interest for Chile. Therefore, the frequency of screening used in the base case was modified from every three years to five years in the vaccination scenario.
- Introduction of the HPV-16/18 AS04-adjuvanted vaccine without cross-protection, or with a cross-protective vaccine efficacy of 45.7% (i.e. lower limit of the 95% CI of cross protection against cervical cancer [68.4%; 95% CI: 45.7 - 82.4]) due to the other oncogenic HPV types [30, 31]) was evaluated.
- Reduced vaccine prices (both vaccines) according to the PAHO 2012 Revolving Fund (13.48 US dollars per dose for the HPV-16/18 AS04-adjuvanted vaccine and 14.25 US dollars per dose for the HPV-6/11/16/18 vaccine) were considered.
- The effects of waning of vaccine efficacy against different HPV types used in the base case analysis were assessed in different scenarios. Waning of vaccine efficacy against HPV-18 and the other ten oncogenic HPV types (overall ~30.0% of cervical cancer cases) was assumed to begin 20 years after vaccination and after five years of a linear decrease in vaccine efficacy this value was assumed to be negligible. This scenario of waning vaccine efficacy was evaluated for vaccination with and without a booster vaccine dose 21 years after the first dose (33 years of age) administered to 95% of the cohort and assumed to result in lifetime protection [46]. Although duration of vaccine efficacy was demonstrated for 9.4 years (Naud et al. 2014) [21] and neutralizing antibody levels are projected to last more than 20 years (Naud et al. 2014 [21], David MP et al. 2009 [39]) waning scenarios against HPV 18 and cross protected HPV types were analyzed based on the results of the quadrivalent vaccine on HPV 18 (Einstein MH, et al. 2011 [47] and Olsson SE, et al. 2007 [48]) (see Additional file 1 for details). Recent studies have shown an overall vaccine efficacy of 93.2% (95% CI 78.9 - 98.7%) for the HPV-16/18 AS04-adjuvanted vaccine against CIN3+ (grade 3 or worse) cases, irrespective of HPV type in the lesion [49]. Therefore, we have also estimated the number of cervical cancer cases and deaths averted considering a vaccine efficacy of 93.2% reported for the HPV-16/18 AS04-adjuvanted vaccine against CIN3+ in an additional scenario, to show the maximum potential impact of this vaccine.
- A vaccination program based on a two dose schedule was analyzed for the HPV-16/18 AS04-adjuvanted vaccine [41, 42]. The two-dose schedule for the HPV-16/18 AS04-adjuvanted vaccine investigated in the present analysis assumed no difference in vaccine efficacy compared to the three-dose schedule. For this scenario, waning of vaccine efficacy was also considered for some HPV types (excluding HPV type 16 as explained earlier), or for all oncogenic HPV types, with or without a booster dose after 21 years of first dose (33 years of age).

#### Probabilistic sensitivity analyses

To test the robustness of model input data and base case assumptions a probabilistic sensitivity analysis (PSA) was performed to quantify the effect of uncertainty surrounding the model input parameters and assumptions on the final ICER estimates. In total, 10000 replicates were generated from the assigned distribution to produce a distribution of the model’s results. The replicates were plotted on the cost-effectiveness plane. The proportion of replicates in each quadrant of the plane was counted and reported as percentage. The PSA was performed by comparing either the HPV-16/18 AS04-adjuvanted vaccine or the HPV-6/11/16/18 vaccine to screening alone or by comparing the HPV-16/18 AS04-adjuvanted vaccine to the HPV-6/11/16/18 vaccine, under different scenarios. The PSA was performed using the @Risk software (Palisade Corporation, Ithaca, New York, USA).

## Results

### Base case analyses

The model was validated by comparing modelled outcomes with published epidemiological parameters for Chile which were obtained from the latest local epidemiological data [10, 35, 36]. Details on model calibration are shown in Table 2 (see Additional file 1 for details) and are aligned with our previous estimates for Chile [10].

**Table 2:** **Results of calibration process for main model estimations**

	Model estimated	Reported	Reference
Prevalence of oncogenic HPV infections	9.7%	9.1% (15–69 years)	[10, 36]
Prevalence of low risk HPV infections	3.1%	3.7% (15–69 years)	[36]
Incident cases of genital warts	4813	4685	[35]
Incident cases of cervical cancer	1370	1331	[35]
Incident cases of deaths associated with cervical cancer	722	759	[35]

Note: HPV: human papillomavirus.

The most significant health and economic outcomes for the base case analysis are shown in Table 3. The HPV-6/11/16/18 vaccine is projected to provide greater benefits in the number of GW and CIN1 cases averted whereas the HPV-16/18 AS04-adjuvanted vaccine is projected to provide greater benefits in the numbers of CIN2 and CIN3, cervical cancer cases and deaths averted. It is estimated that the HPV-6/11/16/18 vaccine is projected to avert 3368 (70.0%) cases of GW and 3522 cases of CIN1 (56.4%). The HPV-16/18 AS04-adjuvanted vaccine is projected to avert 1063 (60.6%) CIN2 and CIN3 cases, 1172 (85.5%) cervical cancer cases and 618 (85.6%) deaths due to cervical cancer, respectively; whereas the HPV-6/11/16/18 vaccine is projected to avert 873 (49.8%) CIN2 or CIN3 cases, 1069 (78.1%) cervical cancer cases and 564 (78.1%) deaths due to cervical cancer, respectively. These differences are projected to result in a gain of 12959/14973 life-years (LYs)/QALYs and 11813/13770 LYs/QALYs for the HPV-16/18 AS04-adjuvanted vaccine and HPV-6/11/16/18 vaccine, respectively.

**Table 3:** **Outcomes for screening, screening + vaccination (base case) for Chilean girls aged 11 years (undiscounted)**

	N	n (%) ^a^	
	No vaccination ^b^	HPV-16/18 vaccine ^c^	HPV-6/11/16/18 vaccine ^c^
Incident cases of genital warts	4813	4813 *(100%)*	1445 *(30.0%)*
Incident cases of CIN1	6249	3100 *(49.6%)*	2727 *(43.6%)*
Incident cases of CIN23	1754	691 *(39.4% )*	881 *(50.2% )*
Incident cases of cervical cancer	1370	198 *(14.5%)*	300 *(21.9 %)*
Incident cases of cervical cancer deaths	722	104 *(14.4%)*	158 *(21.9 %)*
LY	8725602	8738561	8737415
QALY	8723071	8738044	8736841
Total treatment and follow-up related costs^d^	94208827	39776161 *(42.2%)*	43413777 *(46.1%)*
Screening	24878067	25085155 *(100.8%)*	25102940 *(100.9%)*
CIN1 treatment	10223106	5071376 *(49.6%)*	4461609 *(43.6%)*
CIN23 treatment	2869923	1130504 *(39.4%)*	1441268 *(50.2%)*
Genital warts	396244	396334 *(100.0%)*	118954 *(30.0%)*
Cervical cancer	55841487	8092792 *(14.5 %)*	12289006 *(22.0 %)*
Vaccination costs^d^		7044117	7044117
Net total costs^d^	94208827	46820278 *(49.7%)*	50457894 *(53.6%)*

^a^Percentage of no vaccination scenario; ^b^Screening alone scenario; ^c^Screening plus vaccination scenarios; ^d^All costs are presented in 2010 United States dollars.

Note: CIN: cervical intraepithelial neoplasia; LY: life years; QALY: quality-adjusted life years.

The economic analysis projects that the introduction of any HPV vaccine in Chile would be expected to cost 7.0 million US dollars per year (Table 3). However, the HPV-16/18 AS04-adjuvanted vaccine is estimated to be attributed with an additional total treatment and management cost-savings of 3.6 million US dollars in comparison to the HPV-6/11/16/18 vaccine (at price parity per dose) resulting from the additional reduction in the number of cervical cancer treatments required with the HPV-16/18 AS04-adjuvanted vaccine compared with the HPV-6/11/16/18 vaccine. The projected ICUR and ICER for the HPV-6/11/16/18 vaccine compared with the reference (no vaccination) scenario was 541 US dollars per QALY gained or 726 US dollars per LY saved, respectively, while the projected ICUR and ICER for the HPV-16/18 AS04-adjuvanted vaccine was 116 US dollars per QALY gained or 147 US dollars per LY saved, respectively (Table 4). Although the ICUR and ICER estimates from the deterministic analysis show that the HPV-6/11/16/18 vaccine is dominated by the HPV-16/18 AS04-adjuvanted vaccine the introduction of either of the HPV vaccines would be a highly cost-effective strategy in Chile (below the 1X GDP per capita for the cost-effectiveness threshold was 14278 US dollars). This analysis shows that the addition of HPV-16/18 AS04-adjuvanted vaccine to the current screening program of Chile dominates the HPV-6/11/16/18 vaccine as it is projected to provide 29 additional QALY gains or 60 additional LYs saved at a reduced cost (the HPV-16/18 AS04-adjuvanted vaccine cost 344286 discounted US dollars less than the HPV-6/11/16/18 vaccine) per year. It is also projected that both vaccines would reach a similar ICER compared with screening (around 110 US dollars per QALY gained) if the vaccine price per dose for the HPV-6/11/16/18 vaccine is reduced by 1 US dollar (19 US dollars per dose). The HPV-16/18 AS04-adjuvanted vaccine would then appear even more attractive if only the vaccine effects over cervical cancer (no GW considered) or the PAHO Revolving Fund vaccine prices per dose are considered (PAHO prices are lower for the HPV-16/18 AS04-adjuvanted vaccine).

**Table 4:** **Cost-utility and cost-effectiveness analysis (discounted data)**

a. Cost-utility								
	Total ^a^		Incremental		ICUR ^b^	Incremental		ICUR ^b^
			(vs. previous alternative)		(vs. previous alternative)	(vs. no vaccination)		(vs. no vaccination)
	QALYs	Costs	QALYs	Costs		QALYs	Costs	
Current screening practice	2126963	14272773	-		-	-	-	-
HPV-6/11/16/18 vaccine + current screening	2127782	14715753	819	442981	Dominated	819	442981	541
HPV-16/18 + current screening	2127811	14371468	29	-344286	116	848	98695	116
**b. Cost-effectiveness**								
	**Total** ^**a**^		**Incremental**		**ICER** ^**b**^	**Incremental**		**ICER** ^**b**^
			**(vs. previous alternative)**		**(vs. previous alternative)**	**(vs. no vaccination)**		**(vs. no vaccination)**
	**LYS**	**Costs**	**LYS**	**Costs**		**QALYs**	**Costs**	
Current screening practice	2127239	14272773	-		-	-	-	-
HPV-6/11/16/18 vaccine + current screening	2127849	14715753	610	442981	Dominated	610	442981	726
HPV-16/18 + current screening	2127909	14371468	60	- 344286	147	670	98695	147

^a^Strategies are listed in order of increasing health gains (QALYs). ^b^ICUR & ICER calculations for both vaccines are obtained by comparing to previous alternative and to the reference (no vaccination) scenario. Costs and health outcomes were discounted at an annual rate of 6.0% based on the recommendations of the Planning Ministry of Chile [42].

Note: HPV: human papillomavirus; ICUR: incremental cost-utility ratio; ICER: incremental cost-effectiveness ratio; LYS: Life-years saved; QALY: quality-adjusted life years.

### Probabilistic sensitivity analyses

**Figure 1:**
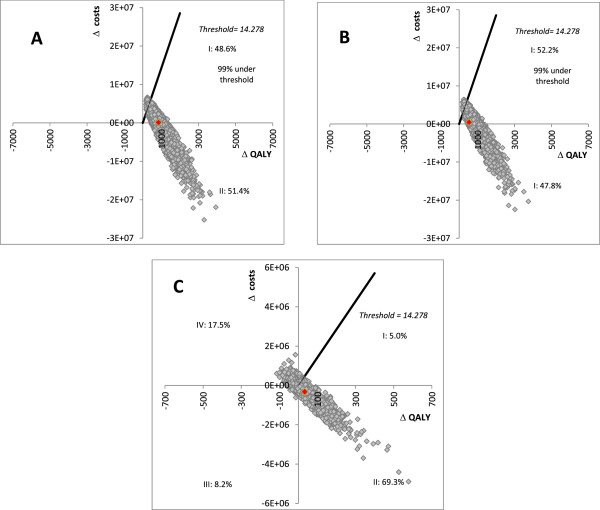
**Probabilistic sensitivity analysis (A) results for the HPV-16/18 vaccine vs. screening as comparator, (6% discount on costs and health outcomes) (B) results for the HPV-6/11/16/18 vaccine vs. screening as comparator (6% discount on costs and health outcomes) and (C) results for the HPV-16/18 vaccine vs. HPV-6/11/16/18 vaccine used as comparator (6% discount on costs and health outcomes).**

The PSA on the ICER of the HPV-16/18 AS04-adjuvanted vaccine (Figure 1A) and HPV-6/11/16/18 vaccine (Figure 1B) versus no vaccination (screening alone) in the base case scenario confirms that both vaccines are highly cost-effective (99.0% of the simulations) and even cost-saving (in 51.4% of the simulations for HPV-16/18 AS04-adjuvanted vaccine and 47.8% for the HPV-6/11/16/18 vaccine). Figure 1C shows that the HPV-16/18 AS04-adjuvanted vaccine have a 69.3% chance to dominate the HPV-6/11/16/18 vaccine although the HPV-16/18 AS04-adjuvanted vaccine has a 8.2% chance to result in less QALY gains with less overall costs than the HPV-6/11/16/18 vaccine and the HPV-6/11/16/18 vaccine has a 17.5% chance to dominate the HPV-16/18 AS04-adjuvanted vaccine. These results are very sensitive to the discount rate. The probability for the HPV-16/18 AS04-adjuvanted vaccine to dominate the HPV-6/11/16/18 vaccine is of 93.0% (undiscounted) and 87.0% with a 3.0% discount rate.

### Deterministic scenario analyses

Table 5 shows the results (cervical cancer cases and deaths, QALYs gained, incremental costs and ICURs) for the different scenarios of interest for both vaccines when compared to screening alone. Considering a lower prevalence of HPV types 16 and 18 (70.0% in cervical cancer cases), the results on cases, deaths, and QALYs gained, incremental costs and ICUR present minimal variation. When a 3.0% discount rate was considered, ICUR was significantly lower than base case and vaccination was projected to be cost-saving. When vaccine introduction was combined with an increase in screening frequency (every five years), a minor reduction in cases and deaths averted was observed with a significant reduction on the incremental costs and the ICUR resulting also in a cost-saving scenario. In contrast, if no cross-protection was included in the analysis for the bivalent vaccine, impact on cervical cancer cases, deaths and QALYs gained was reduced by 17.0% and the incremental costs and ICUR increased significantly (2305 US dollars per QALY gained); however, vaccination is still highly cost-effective. A cross protection vaccine efficacy of 45.7% against cervical cancer resulted in a 6.0% decrease in the vaccine impact on cervical cancer cases, deaths and QALYs gained and the ICUR increased to 589 US dollars per QALY gained. When the PAHO 2012 Revolving Fund vaccine prices were considered both vaccines were projected to be cost-saving but the HPV-16/18 AS04-adjuvanted vaccine saved an additional 615484 US dollars. The scenario without considering efficacy to HPV types 6 and 11 for the HPV-6/11/16/18 vaccine showed a slight decrease in QALYs gained but a significant increase in the incremental costs and ICER (still highly cost-effective). Waning of vaccine efficacy starting 20 years after vaccination showed a small reduction on vaccine impact (6.0–21.0% cases/deaths averted and QALYs gained) with an increase observed in the incremental costs and improvement in the cost-effectiveness profile (715 US dollars per QALY gained; but still highly cost-effective). Additionally, if a booster dose is considered 21 years after the first dose, vaccine impact estimates reached approximately the base case results and the ICER increased to 942 US dollars per QALY gained still resulting in a cost-effective scenario for HPV vaccination in Chile. The scenario with only two doses of the HPV-16/18 AS04-adjuvanted vaccine was cost-saving due to a significant reduction (2.3 million US dollars) in the vaccination costs. Waning of vaccine efficacy in the two dose scenarios, slightly modified the ICER with a 6.0% to 18.0% reduction in QALYs gained and cases or deaths averted (if vaccine effectiveness to HPV 16 is not affected by waning) but a 21.0% to 66.0% reduction on QALYs gained and cases or deaths averted if vaccine efficacy against HPV-16 is affected. Nevertheless, the introduction of a booster dose (third dose) at 33 years of age, allows for the recovery of vaccine impact estimates on cases and deaths with an ICUR still showing a cost-saving result (~ -1800 US dollars per QALY gained).

**Table 5:** **Cost-utility analysis for base case and additional scenarios of interest**

Scenarios	Cervical cancer cases/deaths averted ^a^	QALYs gained ^a^	Incremental costs ^b^	ICUR ^a^
Base case HPV-16/18	1172 / 618	848	98695	116
Base case HPV-6/11/16/18	1069 / 564	819	442981	541
HPV-16/18 prevalence; 70% cervical cancer cases due HPV types 16 and 18^d^	1129 / 595	816	310450	381
HPV-6/11/16/18 prevalence; 70% cervical cancer cases due HPV types 16 and18^d^	977 / 516	751	887321	1181
HPV-16/18 with discounting 3%^e^	1172 / 618	3124	-10198541	dominant^c^
HPV-16/18 + regular screening (every 5 years interval)^f^	1158 / 612	833	-1970016	dominant^c^
HPV-16/18 with no cross-protection^g^	976 / 515	703	1620610	2305
HPV-16/18 with cross-protection assumed as the lower value of 95% CI to cervical cancer (68.4%; 95% CI: 45.7 - 82.4%) [30, 31]^g^	1106 / 583	799	470344	589
HPV-16/18 price as in PAHO 2012 revolving fund^h^	1172 / 618	848	-2197687	dominant^c^
HPV-6/11/16/18 vaccine price as in PAHO 2012 revolving fund^h^	1069 / 564	819	-1582203	dominant^c^
HPV-6/11/16/18 vaccine with no VE to HPV types 6 and11^i^	1069 / 564	772	894820	1159
HPV-16/18 waning (HPV-18 & other oncogenic HPV) after 20 years of first dose /during 5y^j^	955 / 487	794	568274	715
HPV-16/18 waning (HPV-18 & other oncogenic HPV) after 20 years of 1^st^ dose/during 5y + booster dose (21 years after first dose) ^j, k^	1170 / 617	846	797020	942
HPV-16/18, 2 dose vaccine schedule^l^	1172 / 618	848	-2249344	dominant^c^
HPV-16/18 (2 doses) waning (HPV-18 & other oncogenic HPV) after 20 years of 1^st^ dose/during 5y^j, l^	955 / 487	794	-1779765	dominant^c^
HPV-16/18 (2 doses) waning (all HPV) after 20 years of first dose/during 5y ^j, l^	501 / 212	681	-791887	dominant^c^
HPV-16/18 (2 doses) waning (HPV-18 & other oncogenic HPV) after 20 years of first dose/during 5y + booster dose (21 years after first dose^j, k, l^	1170 / 617	846	-1551019	dominant^c^
HPV-16/18 (2 doses) waning (all oncogenic HPV) after 20 years of first dose/during 5y + booster dose (21 years after first dose)^j, k, l^	1164 / 615	841	-1507808	dominant^c^

^a^Compared to no vaccination scenario; ^b^Additional costs compared to no vaccination scenario; ^c^ICUR is showing a cost-saving result; ^d^Base Case: 80% prevalence; ^e^Base case: 6% discount; ^f^Base case: Every 3 years interval; ^g^Base case: CIN2/3 cross protection for bivalent vaccine 68.4% and quadrivalent vaccine 32.5%; ^h^Base case: vaccine cost per dose 20 US dollars for both vaccines –price parity-; ^i^Base case: 98% vaccine efficacy; ^j^Base case: Lifetime duration of vaccine efficacy; ^k^Base case: No booster dose; ^l^Base case: 3 doses.

Note: HPV: human papillomavirus; ICUR: incremental cost-utility ratio; VE: vaccine efficacy; PAHO: Pan American Health Organization; QALY: quality-adjusted life years.

Finally, when an overall vaccine efficacy for the HPV-16/18 AS04-adjuvanted vaccine against CIN3+ cases (irrespective of HPV type in the lesion) of 93.2% is considered the potential impact of the HPV-16/18 AS04-adjuvanted vaccine (Table 6) is greater than our modelled estimates and is projected to avert additional 38 cervical cancer cases and 20 cervical cancer related-deaths than those estimated by the model.

**Table 6:** **Averted cervical cancer cases and deaths based on different estimation methods**

Number of cases and deaths in each scenario	Model estimated			Calculated
	*(based on type-specific VE)*			*(based on 93% VE to CIN3 +* [47]*)*
	No vaccine	HPV-16/18	HPV-6/11/16/18	HPV-16/18
***95% Vaccine coverage***				
Cervical cancer cases	1370	198	300	160
*(% of total cervical cancer)*	*(100.0%)*	*(14.4%)*	*(21.9%)*	*(11.7%)*
Cervical cancer deaths	722	104	158	84
*(% of total cervical cancer deaths)*	*(100.0%)*	*(14.4%)*	*(21.9%)*	*(11.7%)*
***80% Vaccine coverage***				
Cervical cancer cases	1370	406	489	351
*(% of total cervical cancer)*	*(100.0%)*	*(29.6%)*	*(35.7%)*	*(25.6%)*
Cervical cancer deaths	722	213	257	185
*(% of total cervical cancer deaths)*	*(100.0%)*	*(29.5%)*	*(35.6%)*	*(25.6%)*

Note: HPV: human papillomavirus; CIN3+ - cervical intraepithelial neoplasia type 3; VE: vaccine efficacy.

## Discussion

This study broadens the findings of a previous cost-effective analysis of the HPV-16/18 AS04-adjuvanted vaccine for Chile [10]. The analysis shows that the introduction of HPV vaccination with either of the two HPV vaccines is likely to prove highly cost-effective for Chile at 20 US dollars per dose. Even in the worst case scenarios analyzed (reduced prevalence of HPV types 16 and 18 in cervical cancer, no cross-protection, waning of vaccine efficacy) both vaccines were highly cost-effective for Chile. However, addition of the HPV-16/18 AS04-adjuvanted vaccine dominated the addition of HPV-6/11/16/18 vaccine (with a cost-saving of 47 million US dollars when compared to screening alone [undiscounted]) to the existing screening program of Chile. This is an important finding for Chile where a well-organized and successful cervical cancer prevention program (which guarantees the access to diagnosis, treatment, rehabilitation, follow-up and palliative care of cervical cancer cases) was developed under the Explicit Health Warranties reform introduced in 2003 [12].

In the alternative scenarios analyzed, even better health and economic outcomes than the base case scenario are estimated for Chile. When the discount rate was reduced to 3.0% (in-line with international guidelines) or vaccine price was reduced to present 2012 PAHO Revolving Fund vaccine prices or screening interval duration was increased to every five years together with the vaccination program, or a two-dose (versus three dose) vaccination schedule for the HPV-16/18 AS04-adjuvanted vaccine was evaluated, the implementation of cervical cancer vaccination with the HPV-16/18 AS04-adjuvanted vaccine was cost-saving for Chile.

One of the many strengths of this analysis is the comparison of the cost-effectiveness profiles of the HPV-16/18 AS04-adjuvanted vaccine and HPV-6/11/16/18 vaccine for the prevention of cervical cancer updated with recent estimates of clinical, economic and epidemiological data for Chile. The ICURs for the HPV-16/18 AS04-adjuvanted vaccine (116 US dollars per QALY gained) were dominant compared with the HPV-6/11/16/18 vaccine (541 US dollars per QALY gained) in the base case analysis. The PSA confirmed that the HPV-16/18 AS04-adjuvanted vaccine remains dominant over the HPV-6/11/16/18 vaccine (base case scenario) in 69.3% of the simulations generated and this difference was strengthened at lower discount rates (87.0% probability at 3.0% discount as used in other developed countries). Since we assumed similar vaccine efficacies against HPV types 16 and 18 for both vaccines, the difference was based mainly on its different cross-protection vaccine efficacy profiles. In addition, greater benefits for the HPV-16/18 AS04-adjuvanted vaccine can be realized if a 93.2% efficacy against CIN3+ (irrespective of HPV type in the lesion) is considered [49]. Under this scenario, the HPV-16/18 AS04-adjuvanted vaccine was estimated to avert 38 and 20 additional cervical cancer cases and deaths than those based on model type-specific vaccine efficacy estimates, generating an even greater difference in vaccine impact between both vaccines. In this scenario, the HPV-16/18 AS04-adjuvanted vaccine would prevent 88.3% of all cervical cancer cases and deaths while the HPV-6/11/16/18 vaccine would avert 78.1% cervical cancer cases and deaths generating a difference of 140 and 74 cervical cancer cases and deaths prevented per vaccinated cohort of Chilean women between the two HPV vaccines.

The findings from the present analysis are valid and robust mainly due to the inclusion of many plausible scenarios of interest. There is clinical evidence supporting that a two-dose schedule might be possible [40, 41], however further evidence on sustainability of vaccine efficacy is necessary to prepare more robust simulations. Such a scenario would make HPV vaccination cost-saving in Chile while also potentially improving coverage and hence public health benefit at the population level. Nevertheless, waning of vaccine efficacy has the potential to significantly reduce vaccine benefits if it affects the efficacy against HPV 16. If that is the case, we have also investigated the effect of a booster dose (third dose). The booster dose has the potential to recover the benefits identified for the three-dose schedule maintaining the cost-saving outcome for the overall schedule of two-doses with a HPV vaccine booster dose.

The present study shows a much better cost effectiveness profile for these vaccines than previously reported [10]. This change may be attributed to a significant reduction in the price per dose (aligned with present values for the PAHO Revolving Fund), and recent data showing an increase in the disease burden despite the screening program in place for Chile estimated by Globocan 2008 [1] compared to Globocan 2002 [50] and the inclusion of updated cross-protection profiles based on data from latest clinical trials [28–32]. The new data released by Globocan 2012 for Chile (new estimates are 1441/734 cases and deaths) which although slightly higher than those considered in the study, will improve the cost effectiveness results for the vaccines but will not modify the overall conclusions [51].

A potential limitation of this study is that the real-life effectiveness of these vaccines is unknown or the evidence on the sustainability of vaccine efficacy is limited. While we have attempted to identify publications with the best comparable estimates of cross-protection between vaccines, there is no single trial that directly compares the cross protective efficacy of the HPV-16/18 AS04-adjuvanted and the HPV-6/11/16/18 vaccines. In addition, modelling studies based on vaccine efficacy after 6.4 years of follow-up for the HPV-16/18 AS04-adjuvanted vaccine predicted a long-term (≥20 years) persistence of vaccine-induced antibody levels [39]. Although, there is no defined correlate of protection between antibody levels and vaccine efficacy, the latest evidence has shown sustained efficacy after 9.4 years of follow-up [21]. Present estimates provide evidence of sustainability of vaccine efficacy for decades, however, if vaccine efficacy wanes as assumed in the present analysis, a booster dose would be required subsequent to completion of the vaccination schedule. The analysis of waning vaccine efficacy scenario with a booster dose have shown that even in this worst case scenario, the vaccination program will be highly cost-effective (three-dose schedule + booster dose).

Urgent actions are needed in order to reduce cervical cancer mortality. Both interventions (vaccination and screening) must be considered as complementary. Our results show that the inclusion of vaccination against cervical cancer to the current screening program is highly cost-effective or even cost-saving for Chile. The model predicts a substantial impact of the vaccination program not only against cervical cancer cases and deaths but also on pre-cancerous lesions and its associated resource use (diagnostic tests and treatment). However, controversy still exists regarding the best combination of prevention (primary and secondary) programs against cervical cancer and the affordability of such programs. A recent study based on an optimization model have shown that the best combination for Brazil in the prevention of cervical cancer cases under budget constraints was to combine universal HPV vaccination with an increase in screening interval [52]. We have analyzed the scenario of vaccine implementation (base case analysis) together with an extension of screening interval to five years and found no significant difference in the impact of the cervical cancer prevention program in the prevention of cervical cancer cases but these combined interventions are projected to be cost-saving for Chile. Policy makers need to be informed of the progress of scientific evidence in order to decide the best option available for each setting [7, 53]. The present study is aimed to provide additional evidence on the implementation of HPV routine vaccination in a Latin American country like Chile whose present secondary prevention program has attained reasonable success in controlling cervical cancer. The present Chilean scenario with reduced potential benefits for the vaccination program when compared to other countries in the region limits the potential capabilities of a vaccination program to have a good cost-effective profile. However, data from this modelling evaluation favor the addition of HPV vaccination to the current cervical screening program of Chile.

## Conclusions

The modelling exercise conducted assessed that the implementation of HPV vaccination together with the current screening program of Chile would be cost-effective. These findings may further support or guide the introduction of HPV vaccination in other countries of the LAC region where a higher burden of cervical cancer exists.

## Electronic supplementary material

Additional file 1: **Details on the cross protection scenario used, on vaccine efficacy waning scenarios analyzed, on the derivation of HPV incidence from HPV prevalence and on the model simulated age distribution of different outcomes.** (DOCX 200 KB)

## Abbreviations

CIN: Cervical intraepithelial neoplasia
GDP: Gross domestic product
GW: Genital warts
HPV: Human papilloma virus
ICER: Incremental cost-effectiveness ratio
LAC: Latin America and Caribbean region
LY: Life years
Pap: Papanicolaou’s
PAHO: Pan American Health Organization
PSA: Probabilistic sensitivity analysis
QALY: Quality adjusted life years
US: United States.

## References

[1] Ferlay J, Shin HR, Bray F, Forman D, Mathers C, Parkin DM (2010). GLOBOCAN 2008 v1.2, Cancer Incidence and Mortality Worldwide: IARC Cancer Base No. 10 [Internet].

[2] Smith JS, Lindsay L, Hoots B, Keys J, Franceschi S, Winer R, Clifford GM (2007). Human papillomavirus type distribution in invasive cervical cancer and high-grade cervical lesions: a meta-analysis update. Int J Cancer.

[3] World Health Organisation (2009). WHO position paper: human papillomavirus vaccines. Wkly Epidemiol Rec.

[4] Almonte M, Murillo R, Sanchez GI, Jeronimo J, Salmeron J, Ferreccio C, Lazcano-Ponce E, Herrero R (2010). New paradigms and challenges in cervical cancer prevention and control in Latin America. Salud Publica Mex.

[5] Murillo R, Almonte M, Pereira A, Ferrer E, Gamboa OA, Jerónimo J, Lazcano-Ponce E (2008). Cervical cancer screening programs in Latin America and the Caribbean. Vaccine.

[6] Franco EL, Tsu V, Herrero R, Lazcano-Ponce E, Hildesheim A, Munoz N, Murillo R, Sanchez GI, Andrus JK (2008). Integration of human papillomavirus vaccination and cervical cancer screening in Latin America and the Caribbean. Vaccine.

[7] Suárez E, Prieto M (2006). Cervical cancer: the Chilean perspective. FIGO 26^th^ annual report on the results of treatment in gynecological cancer. Int J Gynaecol Obstet.

[8] Sepúlveda C, Prado R (2005). Effective cervical cytology screening programmes in middle-income countries: the Chilean experience. Cancer Detect Prev.

[9] Pan American Health Organization (2010). Report of the Latin American Sub regional Meeting on Cervical Cancer. New Technologies for Cervical Cancer Prevention and Control.

[10] Colantonio L, Gómez JA, Demarteau N, Standaert B, Pichón-Rivière A, Augustovski F (2009). Cost-effectiveness analysis of a cervical cancer vaccine in five Latin American countries. Vaccine.

[11] Pan American Health Organization (2011). PAHO revolving fund: vaccine and syringe prices, 2011. Immun Newsl.

[12] Suárez E, Smith JS, Bosch FX, Nieminen P, Chen CJ, Torvinen S, Demarteau N, Standaert B (2008). Cost-effectiveness of vaccination against cervical cancer: a multi-regional analysis assessing the impact of vaccine characteristics and alternative vaccination scenarios. Vaccine.

[13] Anonychuk AM, Bauch CT, Merid MF, Van Kriekinge G, Demarteau N (2009). A cost-utility analysis of cervical cancer vaccination in preadolescent Canadian females. BMC Public Health.

[14] Debicki D, Ferko N, Demarteau N, Gallivan S, Bauch C, Anonychuk A, Mantovani L, Capri S, Chou CY, Standaert B, Annemans L (2008). Comparison of detailed and succinct cohort modelling approaches in a multi-regional evaluation of cervical cancer vaccination. Vaccine.

[15] Demarteau N, Detournay B, Tehard B, El Hasnaoui A, Standaert B (2011). A generally applicable cost-effectiveness model for the evaluation of vaccines against cervical cancer. Int J Public Health.

[16] Jit M, Demarteau N, Elbasha E, Ginsberg G, Kim J, Praditsitthikorn N, Sinanovic E, Hutubessy R (2011). Human papillomavirus vaccine introduction in low-income and middle-income countries: guidance on the use of cost-effectiveness models. BMC Med.

[17] Demarteau N, Standaert B (2010). Modelling the economic value of cross- and sustained-protection in vaccines against cervical cancer. J Med Econ.

[18] National Institute of Statistics (2004). 1990–2020 Population Projection and Estimations by Age and Sex in Chile.

[19] López MJ, Ili GCG, Brebi MP, García MP, Capurro VI, Guzmán GP, Suárez PE, Ojeda FJM, Roa SJC (2010). Detección y tipificación de virus papiloma humano en lesiones preneoplásicas de cuello uterino. Rev Med Chile.

[20] Ministry of Health (2011). National Health Survey. Chile, 2009–2010.

[21] Naud PS, Roteli-Martins CM, De Carvalho NS, Teixeira JC, de Borba PC, Sanchez N, Zahaf T, Catteau G, Geeraerts B, Descamps D (2014). Sustained efficacy, immunogenicity, and safety of the HPV-16/18 AS04-adjuvanted vaccine: Final analysis of a long-term follow-up study up to 9.4 years post-vaccination. Hum Vaccin Immunother.

[22] Ministry of Health (2010). Cervical cancer clinical guidelines 2010.

[23] Roa JC, Garcia P, Gomez J, Fernández W, Gaete F, Espinoza A, Lepetic A, Suarez E (2009). HPV genotyping from invasive cervical cancer in Chile. Int J Gynaecol Obstet.

[24] Aubin F, Prétet JL, Jacquard AC, Saunier M, Carcopino X, Jaroud F, Pradat P, Soubeyrand B, Leocmach Y, Mougin C, Riethmuller D, EDiTH Study Group: **Human papillomavirus genotype distribution in external acuminata condylomata: a large French National Study (EDiTH IV).** *Clin Infect Dis* 2008,**47**(5)**:**610–615. 10.1086/590560 10.1086/590560 18637758

[25] Paavonen J, Naud P, Salmerón J, Wheeler CM, Chow SN, Apter D, Kitchener H, Castellsague X, Teixeira JC, Skinner SR, Hedrick J, Jaisamrarn U, Limson G, Garland S, Szarewski A, Romanowski B, Aoki FY, Schwarz TF, Poppe WA, Bosch FX, Jenkins D, Hardt K, Zahaf T, Descamps D, Struyf F, Lehtinen M, Dubin G, HPV PATRICIA Study Group (2009). Efficacy of human papillomavirus (HPV)-16/18 AS04-adjuvanted vaccine against cervical infection and pre-cancer caused by oncogenic HPV types (PATRICIA): final analysis of a double-blind, randomised study in young women. Lancet.

[26] Romanowski B, de Borba PC, Naud PS, Roteli-Martins CM, De Carvalho NS, Teixeira JC, Aoki F, Ramjattan B, Shier RM, Somani R, Barbier S, Blatter MM, Chambers C, Ferris D, Gall SA, Guerra FA, Harper DM, Hedrick JA, Henry DC, Korn AP, Kroll R, Moscicki AB, Rosenfeld WD, Sullivan BJ, Thoming CS, Tyring SK, Wheeler CM, Dubin G, Schuind A, GlaxoSmithKline Vaccine HPV-007 Study Group (2009). Sustained efficacy and immunogenicity of the human papillomavirus (HPV)-16/18 AS04-adjuvanted vaccine: analysis of a randomized placebo-controlled trial up to 6.4 years. Lancet.

[27] Future II Study Group (2007). Quadrivalent vaccine against human papillomavirus to prevent high-grade cervical lesions. N Engl J Med.

[28] Brown DR, Kjaer SK, Sigurdsson K, Iversen OE, Hernandez-Avila M, Wheeler CM, Perez G, Koutsky LA, Tay EH, Garcia P, Ault KA, Garland SM, Leodolter S, Olsson SE, Tang GW, Ferris DG, Paavonen J, Steben M, Bosch FX, Dillner J, Joura EA, Kurman RJ, Majewski S, Muñoz N, Myers ER, Villa LL, Taddeo FJ, Roberts C, Tadesse A, Bryan J (2009). The impact of quadrivalent human papillomavirus (HPV; types 6, 11, 16, and 18) L1 virus-like particle vaccine on infection and disease due to oncogenic nonvaccine HPV types in generally HPV-naive women aged 16–26 years. J Infect Dis.

[29] Wheeler CM, Castellsagué X, Garland SM, Szarewski A, Paavonen J, Naud P, Salmerón J, Chow SN, Apter D, Kitchener H, Teixeira JC, Skinner SR, Jaisamrarn U, Limson G, Romanowski B, Aoki FY, Schwarz TF, Poppe WA, Bosch FX, Harper DM, Huh W, Hardt K, Zahaf T, Descamps D, Struyf F, Dubin G, Lehtinen M, HPV PATRICIA Study Group (2012). Cross-protective efficacy of HPV-16/18 AS04-adjuvanted vaccine against cervical infection and pre-cancer caused by non-vaccine oncogenic HPV types: 4-year end-of-study analysis of the randomised, double-blind PATRICIA trial. Lancet Oncol.

[30] Skinner SR, Apter D, Chow SN, Wheeler C, Dubin G, for the HPV PATRICIA Study Group (2009). Cross-protective efficacy of *Cervarix*™ against oncogenic hpv types beyond hpv-16/18: final analysis of cross-protection - PATRICIA study. International Papillomavirus Conference and Clinical Workshop.

[31] Szarewski A (2010). HPV vaccine: cervarix. Expert Opin Biol Ther.

[32] Tjalma W (2009). Efficacy of the HPV-16/18 AS04-adjuvanted vaccine against abnormal cytology and low-grade histopathological lesions in an oncogenic HPV-naïve population. 16th International meeting of the European Society of Gynaecological Oncology (ESGO).

[33] Garland SM, Hernandez-Avila M, Wheeler CM, Perez G, Harper DM, Leodolter S, Tang GW, Ferris DG, Steben M, Bryan J, Taddeo FJ, Railkar R, Esser MT, Sings HL, Nelson M, Boslego J, Sattler C, Barr E, Koutsky LA (2007). Females United to Unilaterally Reduce Endo/Ectocervical Disease (FUTURE) I Investigators: Quadrivalent vaccine against human papillomavirus to prevent anogenital diseases. N Engl J Med.

[34] Muñoz N, Kjaer SK, Sigurdsson K, Iversen OE, Hernandez-Avila M, Wheeler CM, Perez G, Brown DR, Koutsky LA, Tay EH, Garcia PJ, Ault KA, Garland SM, Leodolter S, Olsson SE, Tang GW, Ferris DG, Paavonen J, Steben M, Bosch FX, Dillner J, Huh WK, Joura EA, Kurman RJ, Majewski S, Myers ER, Villa LL, Taddeo FJ, Roberts C, Tadesse A (2009). Impact of human papillomavirus (HPV)-6/11/16/18 vaccine on all HPV-associated genital diseases in young women. J Natl Cancer Inst.

[35] Ministry of Health (2011). Systematization of information cervical cancer in Chile: Review and analysis of the cost-effectiveness studies on HPV vaccines.

[36] Ferreccio C, Prado RB, Luzoro AV, Ampuero SL, Snijders PJ, Meijer CJ, Vaccarella SV, Jara AT, Puschel KI, Robles SC, Herrero R, Franceschi SF, Ojeda JM (2004). Population-based prevalence and age distribution of human papillomavirus among women in Santiago. Chile Cancer Epidemiol Biomarkers Prev.

[37] Monsonego J, Breugelmans JG, Bouee S, Lafuma A, Benard S, Remy V (2007). Anogenital warts incidence, medical management and costs in women consulting gynaecologists in France. Gynecol Obstet Fertil.

[38] XE Corporation: *XE Currency Data Feed Service*. [http://www.xe.com/ict/?basecur=USD&historical=true&month=12&day=28&year=2010&sort_by=name&image.x=42&image.y=17]

[39] David MP, Van Herck K, Hardt K, Tibaldi F, Dubin G, Descamps D, Van Damme P (2009). Long-term persistence of anti-HPV-16 and -18 antibodies induced by vaccination with the AS04-adjuvanted cervical cancer vaccine: modeling of sustained antibody responses. Gynecol Oncol.

[40] Romanowski B, Schwarz TF, Ferguson LM, Peters K, Dionne M, Schulze K, Ramjattan B, Hillemanns P, Catteau G, Dobbelaere K, Schuind A, Descamps D (2011). Immunogenicity and safety of the HPV-16/18 AS04-adjuvanted vaccine administered as a 2-dose schedule compared with the licensed 3-dose schedule: results from a randomized study. Hum Vaccin.

[41] Kreimer AR, Rodriguez AC, Hildesheim A, Herrero R, Porras C, Schiffman M, González P, Solomon D, Jiménez S, Schiller JT, Lowy DR, Quint W, Sherman ME, Schussler J, Wacholder S, CVT Vaccine Group (2011). Proof-of-principle evaluation of the efficacy of fewer than three doses of a bivalent HPV16/18 vaccine. J Natl Cancer Inst.

[42] Ministry of Planning of Chile (2011). Social prices for the evaluation of social projects.

[43] Sachs JD (2001). Macroeconomics and health: Investing in Health for Economic Development. Report of the Commission on Macroeconomics and Health.

[44] International Monetary Fund (2012). World Economic Outlook Database.

[45] Li N, Franceschi S, Howell-Jones R, Snijders PJ, Clifford GM (2011). Human papillomavirus type distribution in 30,848 invasive cervical cancers worldwide: Variation by geographical region, histological type and year of publication. Int J Cancer.

[46] Moscicki AB, Wheeler CM, Romanowski B, Hedrick J, Gall S, Ferris D, Poncelet S, Zahaf T, Moris P, Geeraerts B, Descamps D, Schuind A (2012). Immune responses elicited by a fourth dose of the HPV-16/18 AS04-adjuvanted vaccine in previously vaccinated adult women. Vaccine.

[47] Einstein MH, Baron M, Levin MJ, Chatterjee A, Fox B, Scholar S, Rosen J, Chakhtoura N, Meric D, Dessy FJ, Datta SK, Descamps D, Dubin G, HPV-010 Study Group (2011). Comparative immunogenicity and safety of human papillomavirus (HPV)-16/18 vaccine and HPV-6/11/16/18 vaccine: follow-up from months 12–24 in a Phase III randomized study of healthy women aged 18–45 years. Hum Vaccin.

[48] Olsson SE, Villa LL, Costa RL, Petta CA, Andrade RP, Malm C, Iversen OE, Høye J, Steinwall M, Riis-Johannessen G, Andersson-Ellstrom A, Elfgren K, von Krogh G, Lehtinen M, Paavonen J, Tamms GM, Giacoletti K, Lupinacci L, Esser MT, Vuocolo SC, Saah AJ, Barr E (2007). Induction of immune memory following administration of a prophylactic quadrivalent human papillomavirus (HPV) types 6/11/16/18 L1 virus-like particle (VLP) vaccine. Vaccine.

[49] Lehtinen M, Paavonen J, Wheeler CM, Jaisamrarn U, Garland SM, Castellsagué X, Skinner SR, Apter D, Naud P, Salmerón J, Chow SN, Kitchener H, Teixeira JC, Hedrick J, Limson G, Szarewski A, Romanowski B, Aoki FY, Schwarz TF, Poppe WA, De Carvalho NS, Germar MJ, Peters K, Mindel A, De Sutter P, Bosch FX, David MP, Descamps D, Struyf F, Dubin G, HPV PATRICIA Study Group (2012). Overall efficacy of HPV-16/18 AS04-adjuvanted vaccine against grade 3 or greater cervical intraepithelial neoplasia: 4-year end-of-study analysis of the randomised, double-blind PATRICIA trial. Lancet Oncol.

[50] Ferlay J, Bray F, Pisani P, Parkin DM (2004). IARC Cancer Base No. 5 Version 2.0. GLOBOCAN 2002: Cancer Incidence, Mortality and Prevalence Worldwide.

[51] World Health Organization (2014). GLOBOCAN 2012: Estimated Cancer Incidence, Mortality, and Prevalence Worldwide in 2012.

[52] Demarteau N, Breuer T, Standaert B (2012). Selecting a Mix of prevention strategies against cervical cancer for maximum efficiency with an optimization program. Pharmacoeconomics.

[53] Rogoza RM, Ferko N, Bentley J, Meijer CJ, Berkhof J, Wang KL, Downs L, Smith JS, Franco EL (2008). Optimization of primary and secondary cervical cancer prevention strategies in an era of cervical cancer vaccination: a multi-regional health economic analysis. Vaccine.

[CR54] The pre-publication history for this paper can be accessed here: http://www.biomedcentral.com/1471-2458/14/1222/prepub

