# Supplementary material for: Health economic analysis of human papillomavirus vaccines in women of Chile: perspective of the health care payer using a Markov model
Source: BMC Public Health. 2014 Nov 26;14:1222. doi: 10.1186/1471-2458-14-1222 (PMC4289178; doi:10.1186/1471-2458-14-1222)
Supplement: Supplementary file 1 — Additional file 1: Details on the cross protection scenario used, on vaccine efficacy waning scenarios analyzed, on the derivation of HPV incidence from HPV prevalence and on the model simulated age distribution of different outcomes. (DOCX 200 KB) [file 12889_2013_7394_MOESM1_ESM.docx]

**Additional File 1**

- 1. **Scenarios for Cross Protection**

There is no study directly comparing the benefits of cross-protection associated with the bivalent and quadrivalent human papillomavirus (HPV) vaccines. However, different literature reviews [1–3] and a previous study assessing vaccine impact [4] have shown that both vaccines offer cross protection against some non-vaccine HPV types in individuals without previous HPV infection. Analysis of the most comparable populations suggests that cross-protective vaccine efficacy estimates against infections and lesions associated with non-vaccine HPV types were higher for the bivalent vaccine than the quadrivalent vaccine [1–3]. It was difficult to select vaccine efficacies against individual non-vaccine types for the analysis because their prevalence does not allow the generation of robust point estimates (they usually have large confidence intervals [CI]). Cross protection to some individual HPV types was not statistically significant or even negative, therefore it was decided not to use cross protection data for individual HPV types. All cross protection data were reviewed and analyzed to define the best comparable parameters for the analysis. Consequently, multitype cross protection data against HPV types 31/33/35/39/45/51/52/56/58 and 59 were used for this study. These specific cross-protection values against the same 10 non-vaccine & oncogenic HPV types beyond HPV 16/18 were reported from the clinical trials of both vaccines. While we strived to identify publications with the best comparable estimates of cross-protection between vaccines, there is no single clinical trial that directly compares the cross protective efficacy of the bivalent and quadrivalent HPV vaccines. Although the most comparable subpopulations were selected, it was not possible to fully account for differences in subpopulation characteristics and study assays because this data was obtained from separate clinical trials.

As described earlier, the specific cross protection efficacy values against the same 10 non-vaccine & oncogenic HPV types beyond HPV 16-18 were reported from the clinical trial of both vaccines:

Quadrivalent HPV vaccine [5]: CIN1+= 23.4 (95%CI: 7.8–36.4); CIN2+=32.5 (95%CI: 6.0–51.9)

Bivalent HPV vaccine [6–8]: CIN1+= 47.7 (95%CI: 28.9–61.9); CIN2+= 68.4 (95%CI: 45.7–82.4)

These results showed statistical significance.

- 1. **Waning of vaccine efficacy analyzed in additional scenarios**

The bivalent HPV vaccine does not show any decline on its vaccine efficacy or its IgG and neutralizing antibody levels against HPV 16 or 18 after 9.4 years [9] and vaccine-induced GMTs were well above titers associated with clearance of natural infection in other studies. Neutralizing antibodies against vaccine types are likely to be a major basis of protection against HPV infection and the correlation between the antibodies measured by enzyme-linked immunosorbent assay (ELISA) and (pseudovirion-based neutralization assay (PBNA) has been previously demonstrated [10]. The recent study from Naud et al., 2014 [9] was the longest follow up study reported for a licensed HPV vaccine. Based on these data [9] the results of the modeling predicted that anti-HPV-16 and anti-HPV-18 antibody levels are expected to remain several folds higher than those associated with natural infection for at least 20 years post-vaccination confirming previous estimates by mathematical models [11]. In addition, a head to head randomized and observer-blind study comparing the immunogenicity of both vaccines in healthy women [12] has shown that both vaccines induced higher anti-HPV-16 neutralizing antibody titers than those observed in women who had cleared natural infection. The results from Naud et al [9], also provide circumstantial evidence that should a booster be needed, this will not occur before a substantial amount of time has elapsed after vaccination which is consistent with previous modeling results [11]. These results form the basis to assume lifelong vaccine protection in the base case scenario and should therefore provide confidence in the duration of protection offered by HPV mass vaccination programs existing in a number of countries around the world. Nevertheless we consider waning of vaccine efficacy against HPV 18 and cross protected HPV types in additional scenarios of interest (Table 5), based on the results shown by the quadrivalent vaccine on HPV 18 antibody titers after 5 years of follow up [13] and after 24 months of follow up [12]. Both studies showed that vaccine-induced anti-HPV-18 neutralizing antibody titers reached levels similar to those observed after natural infection in women who received the quadrivalent vaccine. Therefore, we considered it of relevance and interest to explore in additional scenarios the impact of including waning vaccine efficacy for HPV 18 and cross protected HPV types.

Furthermore, we also included the analysis of a 2 dose vaccination regiment in additional scenarios (Table 5). As the evidence on duration of antibody levels and sustained vaccine efficacy is not so extensive for the 2 dose schedule, we considered not only the analysis of scenarios with the waning of vaccine efficacy against HPV 18 and cross protected HPV types but also the waning of vaccine efficacy against all HPV types (including HPV 16) for this schedule.

- 1. **Derivation of HPV incidence from prevalence of low risk HPV**

The incidence of low-risk HPV infection for Chile was calculated using a similar methodology from a previous study for high-risk HPV for Chile [14]. In this case, the prevalence of low-risk HPV infection reported for Chilean women [15] of different ages was converted to incidence data based on natural mortality rate for Chile and the HPV regression and HPV progression rates. The incidence was derived from the prevalence:

*HPV incidence= HPV prevalence – natural mortality – HPV regression – HPV progression*

This computation was repeated for each age group in the entire population. The mortality, regression and progression rates used for this estimate were those used as input data in the Markov model [14]. The incidence of low risk HPV infection by age used in the model is reported in Table A1.

**Table A1 Incidence of lrHPV by age in Chile (based on [15])**

| **Age** | **Probability of low risk HPV infection** |
| --- | --- |
| 12 | 0.0000 |
| 13 | 0.0136 |
| 14 | 0.0198 |
| 15 | 0.0246 |
| 16 | 0.0280 |
| 17 | 0.0303 |
| 18 | 0.0317 |
| 19 | 0.0323 |
| 20 | 0.0323 |
| 21 | 0.0319 |
| 22 | 0.0311 |
| 23 | 0.0301 |
| 24 | 0.0288 |
| 25 | 0.0275 |
| 26 | 0.0261 |
| 27 | 0.0248 |
| 28 | 0.0234 |
| 29 | 0.0222 |
| 30 | 0.0211 |
| 31 | 0.0200 |
| 32 | 0.0192 |
| 33 | 0.0184 |
| 34 | 0.0178 |
| 35 | 0.0173 |
| 36 | 0.0169 |
| 37 | 0.0167 |
| 38 | 0.0166 |
| 39 | 0.0166 |
| 40 | 0.0167 |
| 41 | 0.0168 |
| 42 | 0.0170 |
| 43 | 0.0173 |
| 44 | 0.0177 |
| 45 | 0.0180 |
| 46 | 0.0184 |
| 47 | 0.0189 |
| 48 | 0.0193 |
| 49 | 0.0198 |
| 50 | 0.0203 |
| 51 | 0.0208 |
| 52 | 0.0213 |
| 53 | 0.0218 |
| 54 | 0.0224 |
| 55 | 0.0230 |
| 56 | 0.0236 |
| 57 | 0.0243 |
| 58 | 0.0250 |
| 59 | 0.0257 |
| 60 | 0.0265 |
| 61 | 0.0274 |
| 62 | 0.0283 |
| 63 | 0.0293 |
| 64 | 0.0303 |
| 65 | 0.0314 |
| 66 | 0.0326 |
| 67 | 0.0337 |
| 68 | 0.0349 |
| 69 | 0.0360 |
| 70 | 0.0371 |
| 71 | 0.0381 |
| 72 | 0,0389 |
| 73 | 0.0395 |
| 74 | 0.0397 |
| 75 | 0.0396 |
| 76 | 0.0390 |
| 77 | 0.0378 |
| 78 | 0.0358 |
| 79 | 0.0329 |
| 80 | 0.0289 |
| 81 | 0.0237 |
| 82 | 0.0170 |
| 83 | 0.0086 |
| 84 | 0.0000 |

- 1. **Derivation of the age-specific model fit to HPV prevalence and cancer incidence for Chile**

We calibrated the model on cervical cancer incidence, cervical cancer mortality and genital warts incidence. The distribution of cancer cases, cancer deaths and genital warts by age groups are presented in Figure A1, A2 and A3, respectively. Although the simulation does not perfectly fit reported cases or deaths by the Ministry of Health due to the simulation of HPV cases and deaths at an older age group than reported cases, this simulation derives conservative estimates of cost and quality-adjusted life years (QALYs) averted due to cancer prevention. This difference is attributed to the fact that the model used in our analysis is following a cohort of women over lifetime. Therefore, the age population pyramid generated by the cohort model is different than the age population pyramid presently reported for the female population in Chile. In conclusion, we consider the fitting adequate for the analysis because we are assuming conservative estimates with lower incidence in women of younger ages which are indeed the ones contributing the most to the overall value of the vaccine.

**Figure A1 Distribution of cervical cancer cases by age in Chile**

Note: CC, cervical cancer; MOH– Ministry of Health; X-axis units– age-groups (years)

**Figure A2 Distribution of cervical cancer deaths by age in Chile**

Note: CC, cervical cancer; MOH– Ministry of Health; X-axis units– age-groups (years)

**Figure A3 Distribution of genital warts by age in Chile**

Note: GW, genital warts; X-axis units– age-groups (years)

**References**

1. Lehtinen M, Dillner J: **Clinical trials of human papillomavirus vaccines and beyond**. *Nat Rev Clin Oncol* 2013, **10** (7):400–10.
2. Malagón T, Drolet M, Boily MC, Franco EL, Jit M, Brisson J, Brisson M: **Cross-protective efficacy of two human papillomavirus vaccines: a systematic review and meta-analysis**. *Lancet Infect Dis* 2012, **12** (10):781–789.
3. Lu B, Kumar A, Castellsagué X, Giuliano AR: **Efficacy and safety of prophylactic vaccines against cervical HPV infection and diseases among women: a systematic review & meta-analysis**. *BMC Infect Dis* 2011, **11**:13.
4. Kavanagh K, Pollock KG, Potts A, Love J, Cuschieri K, Cubie H, Robertson C, Donaghy M: **Introduction and sustained high coverage of the HPV bivalent vaccine leads to a reduction in prevalence of HPV 16/18 and closely related HPV types**. *Br J Cancer*. 2014,**110** (11):2804–2811.
5. Brown DR, Kjaer SK, Sigurdsson K, Iversen OE, Hernandez-Avila M, Wheeler CM, Perez G, Koutsky LA, Tay EH, Garcia P, Ault KA, Garland SM, Leodolter S, Olsson SE, Tang GW, Ferris DG, Paavonen J, Steben M, Bosch FX, Dillner J, Joura EA, Kurman RJ, Majewski S, Muñoz N, Myers ER, Villa LL, Taddeo FJ, Roberts C, Tadesse A, Bryan J, et al: **The impact of quadrivalent human papillomavirus (HPV; types 6, 11, 16, and 18) L1 virus-like particle vaccine on infection and disease due to oncogenic nonvaccine HPV types in generally HPV-naive women aged 16-26 years**. *J Infect Dis 2009*, **199** (7): 926−935.
6. Skinner SR, Apter D, Chow SN, Wheeler C, Dubin G; for the HPV PATRICIA Study Group: **Cross-protective efficacy of Cervarix™ against oncogenic hpv types beyond hpv-16/18: final analysis of cross-protection - PATRICIA study**. Page 67/69. International Papillomavirus Conference and Clinical Workshop, Malmö, Sweden, 8−14 May, 2009.
7. Szarewski A: **HPV vaccine: Cervarix**. *Expert Opin Biol Ther* 2010, **10** (3): 477−487.
8. Tjalma W: **Efficacy of the HPV-16/18 AS04-adjuvanted vaccine against abnormal cytology and low-grade histopathological lesions in an oncogenic HPV-naïve population**. 16th International meeting of the European Society of Gynaecological Oncology (ESGO). Belgrade, Serbia, 11−14 October 2009.
9. Naud PS, Roteli-Martins CM, De Carvalho NS, Teixeira JC, de Borba PC, Sanchez N, Zahaf T, Catteau G, Geeraerts B, Descamps D: **Sustained efficacy, immunogenicity, and safety of the HPV-16/18 AS04-adjuvanted vaccine: Final analysis of a long-term follow-up study up to 9.4 years post-vaccination**. *Hum Vaccin Immunother* 2014, **10** (8):29–28.
10. Dessy FJ, Giannini SL, Bougelet CA, Kemp TJ, David M-PM, Poncelet SM, Pinto LA, Wettendorff MA. **Correlation between direct ELISA, single epitope-based inhibition ELISA and pseudovirionbased neutralization assay for measuring anti-HPV-16 and anti-HPV-18 antibody response after vaccination with the AS04-adjuvanted HPV-16/18 cervical cancer vaccine**. *Hum Vaccin* 2008, 4:425–34.
11. David MP, Van Herck K, Hardt K, Tibaldi F, Dubin G, Descamps D, Van Damme P. **Long-term persistence of anti-HPV-16 and -18 antibodies induced by vaccination with the AS04-adjuvanted cervical cancer vaccine: modeling of sustained antibody responses**. *Gynecol Oncol* 2009, **115** (Suppl):S1–6.
12. Einstein MH, Baron M, Levin MJ, Chatterjee A, Fox B, Scholar S, Rosen J, Chakhtoura N, Meric D, Dessy FJ, Datta SK, Descamps D, Dubin G; HPV-010 Study Group. **Comparative immunogenicity and safety of human papillomavirus (HPV)-16/18 vaccine and HPV-6/11/16/18 vaccine: follow-up from months 12-24 in a Phase III randomized study of healthy women aged 18-45 years***. Hum Vaccin* 2011, **7** (12):1343–58.
13. Olsson SE, Villa LL, Costa RL, Petta CA, Andrade RP, Malm C, Iversen OE, Høye J, Steinwall M, Riis-Johannessen G, Andersson-Ellstrom A, Elfgren K, von Krogh G, Lehtinen M, Paavonen J, Tamms GM, Giacoletti K, Lupinacci L, Esser MT, Vuocolo SC, Saah AJ, Barr E: **Induction of immune memory following administration of a prophylactic quadrivalent human papillomavirus (HPV) types 6/11/16/18 L1 virus-like particle (VLP) vaccine**. *Vaccine* 2007, **25** (26):4931–4939.
14. Colantonio L, Gómez JA, Demarteau N, Standaert B, Pichón-Rivière A, Augustovski F: **Cost-effectiveness analysis of a cervical cancer vaccine in five Latin American countries**. *Vaccine* 2009, **27** (40):5519–5529.
15. Ferreccio C, Prado RB, Luzoro AV, Ampuero SL, Snijders PJ, Meijer CJ, Vaccarella SV, Jara AT, Puschel KI, Robles SC, Herrero R, Franceschi SF, Ojeda JM: **Population-based prevalence and age distribution of human papillomavirus among women in Santiago, Chile**. *Cancer Epidemiol Biomarkers Prev* 2004, **13** (12): 2271–2276.
16. Monsonégo J, Breugelmans JG, Bouée S, Lafuma A, Bénard S, Rémy V: **Anogenital warts incidence, medical management and costs in women consulting gynaecologists in France**. *Gynécologie Obstétrique & Fertilité* 2007, **35** (2): 107–113.
